# 

**Published:** 1852-09

**Authors:** 


### CONTENTS

OF THE

### MEDICAL EXAMINER.

NEW SERIES—NO. XCIII.—SEPTEMBER, 1852.

### ORIGINAL COMMUNICATIONS.

Experimental Researches applied to Physiology and Pathology. By E. Brown-Séquard, M. D., of Paris,

On the Causes of the Torpidity of the Tenrec, - - 549

On the Influence of Poisons upon Animal Heat as a Cause of Death, - - - - - - - 550

On certain Actions of Cold, Warmth and Light upon the Crystalline Lens, - - - - - - 553

On the Normal Degree of the Temperature of Man, - 554

On the Influence exerted upon the General Temperature of the body by a change in the Temperature of one of the Extremities, - - - - - - 556

Action of Cold on the Coagulability of Blood, and Persistence of Life in Frogs after losing half of the Ventricle of the Heart, - - - - - - - 559

On a Singular Case of Animal Graft, - - - 560

On a Convulsive Affection produced by extensive Injuries of the Spinal Cord, - - - - 561

On the relations existing between the Organisation of Nerve Tubes and their Vital Properties, - - 563
On the Persistence of Life in Animals deprived of their Medulla Oblongata, - - - - - 565
On Dysentery. By George S. Upshur, A. M., M. D., Fellow of the Medical Society of Virginia; Member of the American Medical Association, &c., Norfolk, Virginia, - - - 570
Successful Removal of a Foreign Body from the Knee-joint.—Anomalous Character of the same. By J. Washington Smith, M. D., of Croton, Delaware Co., N. Y., - - - - 578
Case of Conception before the Appearance of the Menses.. By Wm. T. Taylor, M. D., Philadelphia, - - - - 581
Case of Fracture of the Cranium. By H. J. Rice, M. D., of Rockville, Indiana, - - - - - - - 582
Ice as a local Anæsthetic. By W. A. Berry, M.D., Washington, D.C., 585

### CLINICAL REPORTS.

Clinic of Jefferson Medical College. Service of Prof. Dunglison. February, 1852. (Reported by J. Aitken Meigs, M. D.,) - 586

### EDITORIAL.

Death of Dr. Isaac Parrish, - - - - - - 593
Appointment in the University of Pennsylvania, - - - 593
Appointments in the University of Louisville, - - - 593
Announcement of a New Medical School in Cincinnati, - - 593

### MEDICAL NEWS.

Proceedings of the Northern Medical Association in relation to the Death of Dr. George W. Patterson, - - - - 593
Letter from Dr. John Hastings, of San Francisco, California, - 594

### RECORD OF MEDICAL SCIENCE.

### PATHOLOGY AND PRACTICE OF MEDICINE.

An Inquiry into the proximate cause of Gout and its rational Treatment. By Anthony White, Esq., M. B., Cambridge, late President of the Royal College of Surgeons of England, - - 595

Three Cases of Paracentesis Thoracis in Pleurisy; two of which were successful. By Dr. Joseph Beyran, - - - - 603

SURGERY.

On the Diagnosis of Chronic Ovarian Tumors. By E. J. Tilt, M. D., Senior Physician to the Farringdon General Dispensary and Lying-in Charity, and to the Paddington Free Dispensary for Diseases of Women and Children, - - - - 604
Entropium proved to depend on Muscular Action. By Haynes Walton, Esq., F. R. C. S., Surgeon to the Central London Ophthalmic Hospital, and to St. Mary Hospital, Paddington, 608
Ligation of the Primitive Iliac Artery, - - - - 611
Mr. Barnard Holt's Stricture Dilator, - - - - 611

CHEMISTRY.

The State in which Oxygen exists in the Blood, - - - 613
Antidote for Cupreous Salts, - - - - - 614
Test for the Presence of Mercury, - - - - - 614
On the Examination of Ointments containing Oxide of Mercury, - 615
On the Hydrated Peroxides of Iron and Magnesia as Antidotes in Poisoning with Arsenic, - - - - - 515

MATERIA MEDICA AND THERAPEUTICS.

On Guarana. By D. Ritchie, Esq., - - - - 616

OBSTETRICS.

Removal of a Pessary after forty-one years' residence in the Pelvis, 616

